# The effects of a state concussion law on the frequency of sport-related concussions as seen in two emergency departments

**DOI:** 10.1186/s40621-015-0034-7

**Published:** 2015-02-26

**Authors:** Thomas Trojian, Pina Violano, Matthew Hall, Charles Duncan

**Affiliations:** 1 Division of Sports Medicine, Drexel University College of Medicine, 10 Shurs Lane, Philadelphia, PA 19127 USA; 2 Trauma Department, Yale-New Haven Hospital, 300 George Street, 4th Floor, Room 443, New Haven, CT 06510 USA; 3 Injury Free Coalition for Kids of New Haven, Yale-New Haven Children’s Hospital, 300 George St 4th Floor, Room 443, New Haven, CT 06510 USA; 4 Department of Orthopaedics, Sports Concussion Program, UCHC, 263 Farmington Avenue, Farmington, CT 06263 USA; 5 Department of Neurosurgery, Yale School of Medicine, 333 Cedar Street, New Haven, CT 06510 USA

**Keywords:** Concussion, Concussion law, CT Public Act 10-62, Head injury, Sport-related concussion

## Abstract

**Background:**

Connecticut (CT) passed its original sport-related concussion (SRC) law (PA 10-62) in 2010. The law requires that a health-care professional evaluate high school athletes with concussion symptoms. The purpose of this study was to evaluate two level 1 Trauma Center Emergency Department (ED) records for SRC before and after the Connecticut Public Act (CT PA) 10-62 to determine if the law had an effect on the presentation to the ED of SRCs.

**Methods:**

A retrospective analysis of two level 1 Trauma Center Emergency Departments database was performed. Monthly data on SRCs treated in the study EDs from July 2003 through June 2012 were collected and analyzed using the autoregressive integrated moving average model. The number of SRCs in the youth (under age 14 years), high school (age 14 to 18 years), and adult (age >18 years) populations prior to CT PA 10-62 was compared to the number of SRCs post implementation of CT PA 10-62 for each academic school year, fall sports season, and summertime.

**Results:**

Monthly SRCs in high school students treated in the study EDs increased from 2.5 cases to 5.9 cases between pre and post implementation of CT PA 10-62 (*p* < 0.001). Statistical modeling revealed that implementation of CT PA 10-62 was associated with significantly increased SRCs treated in the study EDs and that the increase was limited to the high school students in the fall season and during the school year.

**Conclusions:**

There has been a marked increase in the frequency of SRCs treated in the emergency departments in the high school population in Connecticut after the implementation of the sport-related concussion law. The results suggest that the sport-related concussion law in Connecticut is effective in improving the evaluation and detection of SRCs in high school students.

**Electronic supplementary material:**

The online version of this article (doi:10.1186/s40621-015-0034-7) contains supplementary material, which is available to authorized users.

## Background

Concussions are a common occurrence in high school sports (Guerriero et al. 2012; Marar et al. 2012; Meehan and Mannix 2010; Nation et al*.*^2011^). The number of sport-related concussions (SRC) in the past decade being diagnosed is increasing (Guerriero et al*.*^2012^). The exact reason for the increase is not known, and it may be due to increased awareness and knowledge from concussion laws related to youth sports (Trojian and Hoey 2012). As of 2014, all states have passed laws concerning concussions in high school and youth sports (Center for Disease Control and Prevention 2013; National Conference of State Legislatures 2014). These laws require the removal of athletes from play when concussion is suspected, and subsequent evaluated by a licensed medical professional (Tomei et al*.*^2012^).

Connecticut was one of the first states to pass a law mandating the removal of an athlete and evaluation by a health-care professional. The Connecticut Public Act (CT PA) 10-62 An Act Concerning Student Athletes and Concussions went into effect on July 1, 2010 (Anonymous 2010). This law requires that ‘a student athlete be removed from play or other kinds of physical exertion when showing signs of a concussion, and are not permitted to resume participation without written clearance from a licensed medical professional’. It applies to all student athletes participating in Connecticut Interscholastic Athletic Conference (CIAC)-sponsored athletics. CT PA 10-62 includes that ‘The coach of any intramural or interscholastic athletics shall immediately remove a student athlete from participating in any intramural or interscholastic athletic activity who: (A) is observed to exhibit signs, symptoms or behaviors consistent with a concussion following an observed or suspected blow to the head or body, or (B) is diagnosed with a concussion.’ As well, CT PA 10-62 includes that ‘The coach shall not permit such student athlete to participate in any supervised team activities involving physical exertion, including: practices, games or competitions, until such student athlete receives written clearance to participate in such supervised team activities involving physical exertion from a health-care professional trained in the evaluation and management of concussions.’ (Anonymous 2010). The intent of CT PA 10-62 and similar laws across the country is to increase safety and ensure appropriate evaluation and management of SRC. A study in 2011 by Giebel et al*.* showed that ED physicians take into consideration important clinical factors in assessing patients with SRC (Giebel et al*.*^2011^). For instance, a graded symptom checklist completed in the ED reliably identified concussion symptoms for all children aged 6 years and older (Grubenhoff et al*.*^2010^). This was recommended by the fourth Consensus Conference on Concussions (McCrory et al*.*^2013^) and the new American Academy of Neurology (AAN) guidelines for evaluation and management of concussion (Giza et al*.*^2013^).

The implementation of CT PA 10-62 should increase the reported number of diagnosed SRCs. CT PA 10-62 only applies to interscholastic and intramurals athletes at a high school level and would not cover summer sports activities. High school coaches report that they refer almost 40% of their athletes with concussion symptoms to the ED (Williams et al*.*^2012^). Therefore, we would expect an increase in ED SRC visits during the school year in high school athletes.

We hypothesized that the diagnosis of SRC in high school athletes, as defined by the age group 14-18 years old, presenting to the ED would significantly increase after the CT PA 10-62 law was implemented. Since the law applies neither to adults (>18 years) nor youth (less than 14 years) populations, we hypothesized there would be no significant change in the number of SRC diagnosed for these populations pre and post CT PA 10-62. We hypothesize that there would be no increase in SRC during the summer season (July and August) since there are no CIAC sponsored athletic events in Connecticut. This would be the first study to investigate the effect on the medical system of a state law pertaining to SRC.

## Methods

Data were collected in the emergency departments (EDs) of two major academic, level 1 adult and level 1 pediatric, trauma centers as part of the traumatic injury database (TraumaBase, Clinical Data Management, Denver, Colorado) and forwarded to the Connecticut Department of Health. Both trauma centers are verified by the American College of Surgeons and designated by the Department of Public Health in the State of Connecticut. The ED patient volume for the Adult Trauma Center in 2013 was 92,679 with 15,973 having some sort of traumatic (international classification of diseases (ICD)-9 codes 800-959.99) injury, 3,312 of those patients activated the trauma system or had some sort of adult trauma consult. The ED patient volume for the Pediatric Trauma Center in 2013 was 28,558 with 7,105 having some sort of traumatic (ICD-9 codes 800-959.99) injury, 796 of those patients activated the trauma system or had some sort of pediatric trauma consult. These data have been collected since 2003; the de-identified data include the diagnosis of concussion and sport participation. An additional field of sport-related activity was also collected by the trauma department of both hospitals. All medical records of patients in contact with the ED were reviewed (via EPIC patient chart and EPIC reports targeting ICD-9 codes 800-959.99 AND/OR consult with a physician who is part of the trauma service AND/OR activation of the trauma service) by the hospital Trauma Data Coordinator. A patient list was generated which meets the patient criteria (traumatic ICD-9 AND (Activation OR Consult)). This patient list was then disseminated to the Trauma Registry staff, who reviewed patient records within EPIC for pertinent data to the trauma registry, including demographics, injury details, ED and hospital specific data including vital signs, procedures, medications, and consults, as well as ‘stay’ data including nursing unit progression, outcomes, complications, and discharge information. The trauma registry dataset includes a subset of data defined by, and regulated by, the National Trauma Database standards. There are additional data, defined by the institutions and/or State Trauma Committee, also included within the dataset.

The State requires that all facilities accepting trauma patients (defined by ICD-9 code) are required to submit data quarterly to the Department of Public Health. This process involves running an extract from the hospital Trauma Registry software (in most cases, provided by the State of Connecticut), and submitting it to the State, via push button methodology. The state collects demographics, injury information, ED and hospital specific data, as well as ‘stay’ data. A specific Data Dictionary of what is submitted is defined in the CT State Statute 19a-177-7. (http://www.ct.gov/dph/lib/dph/public_health_code/sections/19a-177-1_to_19a-177-9_statewide_trauma_system.pdf).

The Institutional Review Board of the University of Connecticut approved the evaluation of the de-identified data.

The injury database was screened for method of injury = sport, type of injury = blunt, region of injury = head, and abbreviated injury scale (AIS) severity score >= 2. The AIS was set at >=2 to avoid problems with changes in AIS coding that were implemented in 2010 in these ED but released in 2005 (Stewart et al*.*^2011^). Data were divided into ages of <14 years (youth), 14 to 18 years (high school), and over 18 years (adults). Data of monthly SRC diagnoses from July 2003 through June 2013 was available for analysis. CT PA 10-62 applies to CIAC-related sports, which are played during September to June. The data were evaluated in two groups as ‘school year’ (when CT PA 10-62 is applicable), from September to June, and ‘Summer’ (when CT PA 10-62 does *not* apply) including both July and August. Connecticut athletic programs do not start to practice fall sports until the last week of August.

Statistical evaluation of the time trends was performed using the standard deviation method and time series analysis. An autoregressive integrated moving average (ARIMA) model was used. This is a generalization of an ARMA model. These models are fitted to our data since our data were a time series with seasonal variation and a defined time point when an event occurred (Ely et al*.*^1997^; Ottenbacher and York 1984). The date of implementation of CT PA 10-62 (July 2010) was chosen as the event date. IBM SPSS 20 (IBM Corp., Armonk, NY, USA) was used to perform the analysis. Since these data showed significant seasonal trends, seasonal decomposition using the additive method was performed and our data are presented as both raw and seasonal-adjusted data. A time-series analysis was performed since there was an increasing trend over the time of the diagnosis of SRCs due to increase awareness. Adjustment to the *p* value for multiple comparisons was performed using the Dunn-Šidák correction method.

## Results

**Figure 1:**
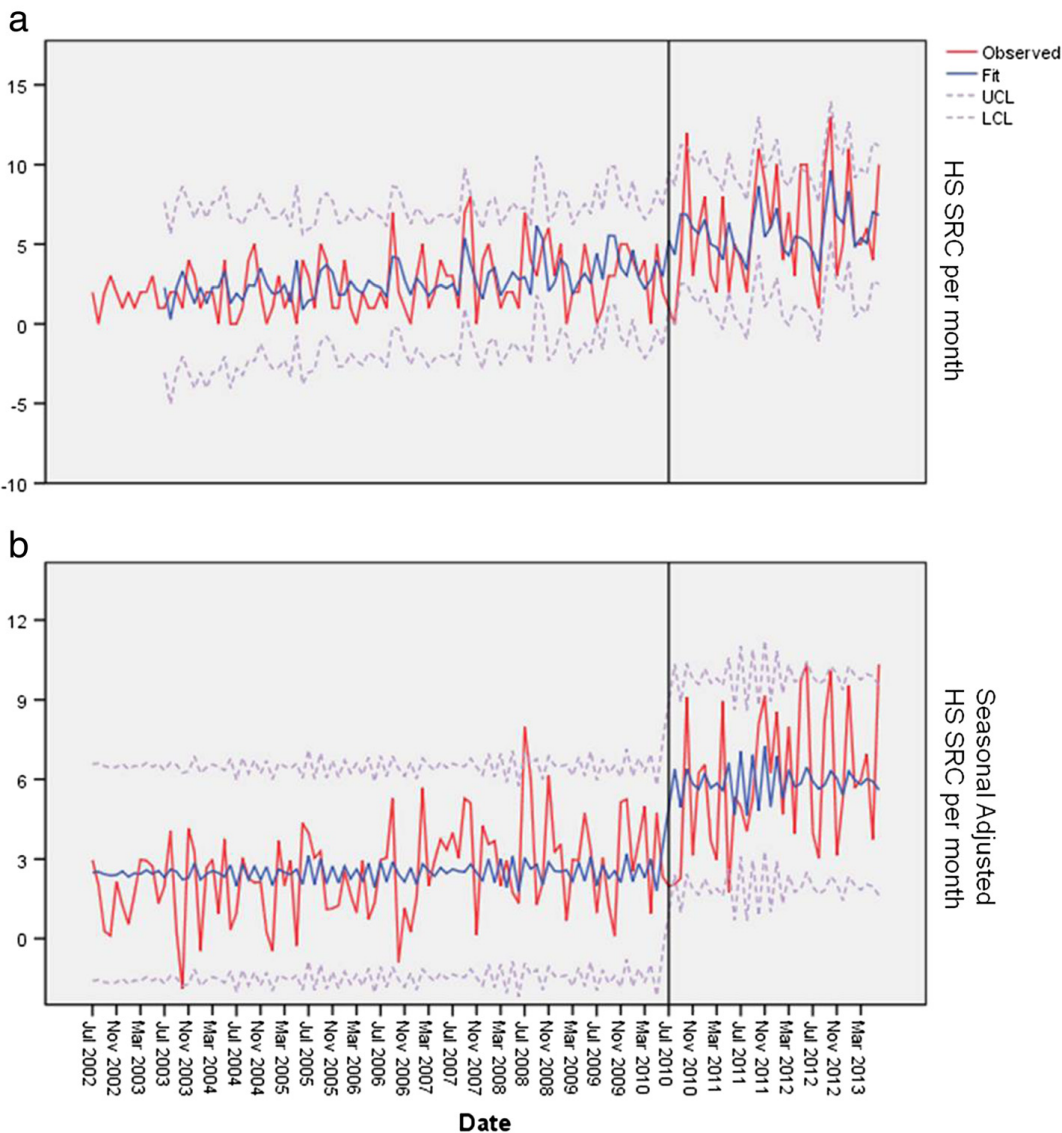
**High school SRC by month.** This is a plot of the high school concussion from September 2002 to June 2013. The line is the fit line plus 2 SD **(a)**. The lower plot is the seasonal-adjusted data **(b)**. *Note: The vertical line indicated the implementation of the law.

**Figure 2:**
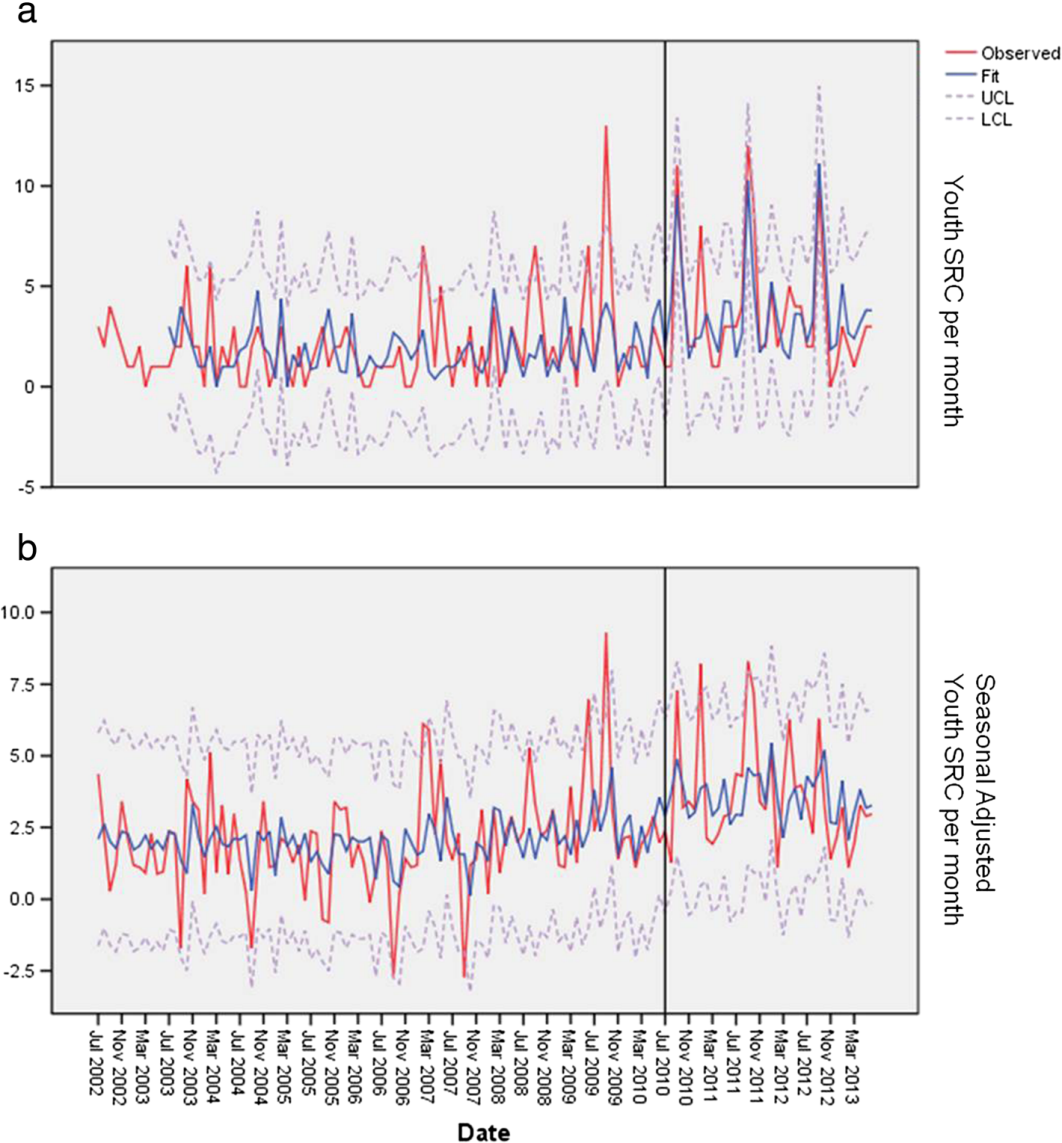
**Youth SRC by month.** This is a plot of the youth concussions plot of the high school concussion from September 2002 to June 2013. The line is the fit line plus 2 SD **(a)**. The lower plot is the seasonal-adjusted data **(b)**. *Note: The vertical line indicated the implementation of the law.

**Figure 3:**
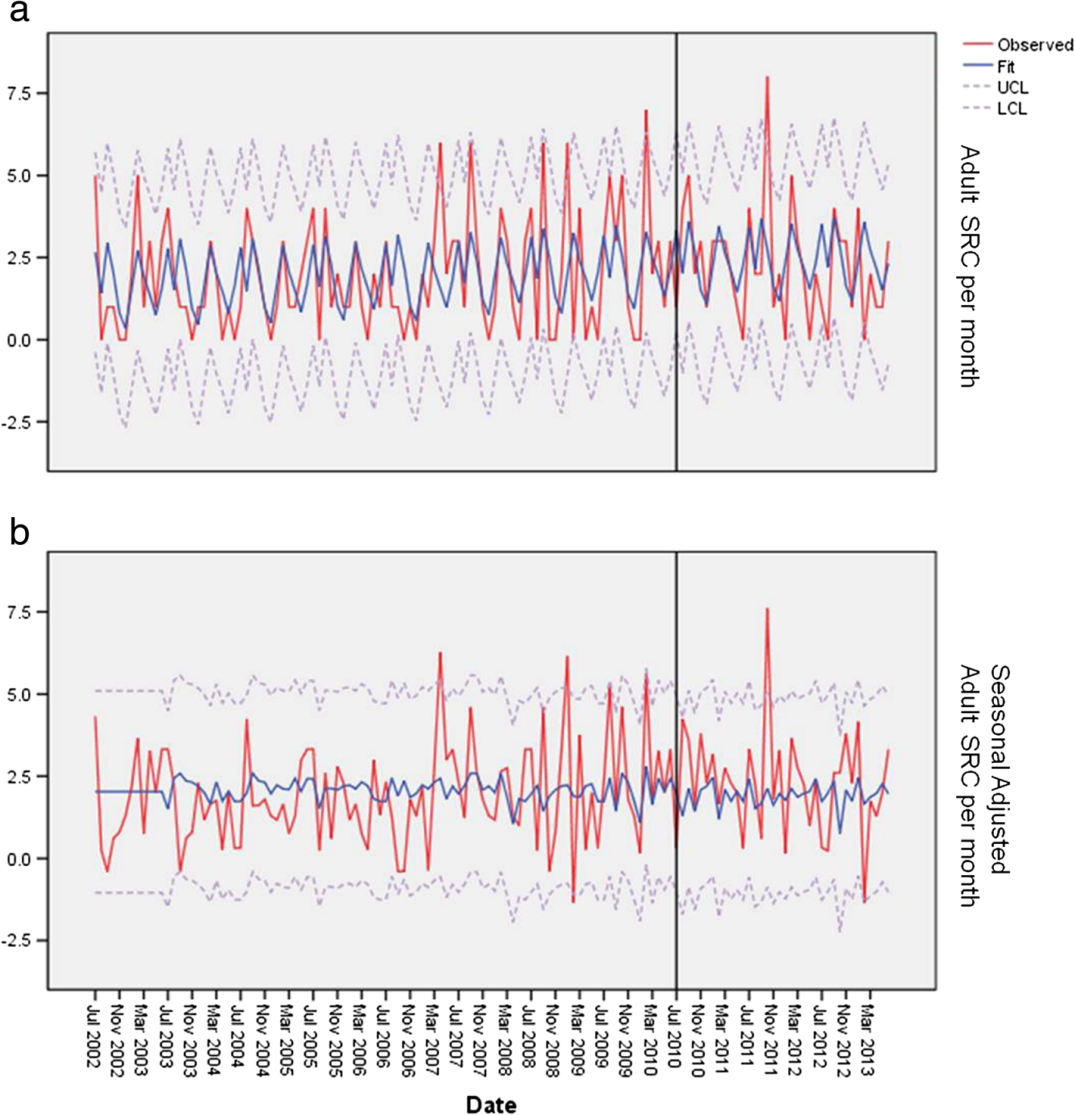
**Adult SRC by month.** This is a plot of the adult concussions plot of the high school concussion from September 2002 to June 2013. The line is the fit line plus 2 SD **(a)**. The lower plot is the seasonal-adjusted data **(b)**. *Note: The vertical line indicated the implementation of the law.

The number of ED visits per month for all pediatric patients that were treated and released averaged 391.4 (SD ± 50.4). The number of visits did not significantly vary by year. The number of SRCs seen in the high school population for the first 96 months immediately prior to CT PA 10-62 was *n* = 238 (mean 2.48 SRC per month, SD ± 1.85), for the 36 months after CT PA 10-62 *n* = 213 SRCs (mean 5.92 SRC per month, SD ± 3.52) (Table 1). The number of SRCs in all of the years prior to CT PA 10-62 compared to the years post CT PA 10-62 during the summer months for high school, youth, or adults was not significantly different for any of the groups. All groups (youth, high school, and adult) showed seasonal trends (Figures 1a, 2a, and 3a). The number of SRCs for high school was significantly different between all of the years prior to CT PA 10-62 compared to the years post CT PA 10-62 (*p* < 0.01) and comparing pre CT PA 10-62 and post CT PA 10-62 (*p* < 0.02) (Figure 1a). Seasonal-adjusted plot for high school showed significant difference between pre and post PA 10-62 (*p* < 0.02) (Figure 1b).

**Table 1:** **Average monthly emergency department visits for sport-related concussions**

**Age group**	**Pre CT PA 10-62**			**Post CT PA 10-62**			**Total**			**Pre/post** ***p*** **value**
	**Total**	**Mean (±SD)**	**95% CI**	**Total**	**Mean (±SD)**	**95% CI**	**Total**	**Mean (±SD)**	**95% CI**	
Youth	195	2.0 (±2.0)	1.6 to 2.4	130	3.6 (±2.9)	2.7 to 4.6	325	2.5 (±2.4)	2.0 to 2.9	0.005
High school	238	2.5 (±1.9)	2.1 to 2.9	213	5.9 (±3.5)	4.8 to 7.1	451	3.4 (±2.9)	2.9 to 3.9	<0.000
Adult	185	1.9 (±1.7)	1.6 to 2.3	82	2.3 (±1.7)	1.7 to 2.8	267	2.0 (±1.7)	1.7 to 2.3	0.5172

The number of SRCs for the youth age group was significantly different from all of the years prior to CT PA 10-62 compared to the years post CT PA 10-62 (*p* < 0.01), but no statistical difference was seen prior to the implementation of CT PA 10-62 and post PA 10-62 (*p* = 0.11) (Figure 2a). Seasonal-adjusted plot of youth SRC was not significant between pre and post CT PA 10-62 (*p* = 0.09) (Figure 2b). The number of SRCs in adults was not significantly different from all of the years prior to CT PA 10-62 compared to the years post CT PA 10-62 (*p* > 0.1), and no statistical difference was seen prior to the implementation of CT PA 10-62 and post PA 10-62 (*p* > 0.1) (Figure 3a,b).

## Discussion

A state concussion law (CT PA 10-62) significantly influenced the ED visits for SRC in the high school age population. The number of SRC in the 14-to-18-year-old age group significantly increased (Figure 1a,b) after the onset of the law. No difference in the rate of ED visits were seen pre and post law in the summer time for any group. There was no significant change in the number of SRCs in the adult population diagnosed over time, which helps support our hypothesis, as the law does not apply to the adult population. For the youth population (<14 years), no significant change was seen in the number of SRC between the dates pre and post CT PA 10-62. The number of SRC in the youth age group has increased steadily over time, but the introduction of the law did not significantly influence this increasing trend. This difference is shown in Table 1 where the average SRC visits changed over time, but this significant change is not seen in the time series by the event of the implementation of the law as seen in Figure 2a,b. The increase over time may represent an increasing awareness about SRC in the youth population. We feel the increase in high school diagnosis of SRCs between pre and post CT PA 10-62 implementation dates demonstrates the effect of a state concussion law resulting in more evaluations of high school student athletes with SRCs in the ED.

Our study shows an increasing number of all pediatric SRCs being diagnosed in the ED. Bakhos et al*.* noted a twofold increase in the number of SRC visits from 1997 to 2007 (Bakhos et al*.*^2010^). In addition, there was an increase in SRCs in the 14-to-18-year-old age group from earlier to later years of our study prior to the implementation of the law then another increase after CT PA 10-62 (Bakhos et al*.*^2010^).

High school football coaches have recommended the law be expanded to youth levels (Trojian and Hoey 2012). The numbers of youth athletes with SRCs presenting to the ED are increasing, but further expansion of the state law to youth sports should be considered. Our study showed that this legislation does not cause a residual increase in presentation of SRCs to the ED in other age groups. We have identified that the law is not affecting summer SRC visits. Changes to state concussion laws could include summer camps, travel teams, and all-star teams to ensure that all children with SRC are getting appropriate care and education. Concussion laws mandating the removal of athletes with a head injury from play might be expanded to include all organized sports at all levels.

Our study has certain limitations. The high school sports season does not match up exactly to the months of the school year. For example, fall sports begin in the last week of August, and therefore, those SRCs would not be covered under CT PA 10-62 and therefore missed in our study count. In addition, our results are limited to only ED visits of one level 1 Pediatric Trauma Center and one level 1 Adult Trauma Center in Connecticut and therefore may not be generalizable to the entire state. The study subjects included individuals with an AIS severity of >=2 which excludes some SRCs with scores of 1. We felt this better represented the data prior to 2005 and post 2005 AIS coding changes and therefore improved reliability of our data. An alternative explanation for our results could be that we found an increase in SRC diagnosed in the EDs only, but the actual number of SRC in the state of Connecticut from sports may not have changed. Therefore, the increase we report may reflect an increase in the percentage of SRCs presenting to the ED after the implementation of the law rather than a true increase in SRC occurrence.

## Conclusions

CT PA 10-62 has contributed to an increased number of high school athletes (but not youth age athletes) being evaluated at two level 1 Trauma Center EDs for SRCs. State concussion laws, such as CT PA 10-62, are intended to increase student athlete safety and to ensure appropriate diagnosis and management of SRCs. The law has increased ED visits for SRC for high school student athletes, and expansion of the law to include youth sports and summer camps should be considered.
